# Combined Inhibitory Effect of Fir (*Abies alba* Mill.) Honeydew Honey and Probiotic Bacteria *Lactiplantibacillus plantarum* on the Growth of *Salmonella enterica* Serotype Typhimurium

**DOI:** 10.3390/antibiotics11020145

**Published:** 2022-01-24

**Authors:** Silvestar Mežnarić, Irena Brčić Karačonji, Goranka Crnković, Andrija Lesar, Tomislav Pavlešić, Darinka Vučković, Ivana Gobin

**Affiliations:** 1 Department of Microbiology and Parasitology, Faculty of Medicine, University of Rijeka, Brace Branchetta 20, 51000 Rijeka, Croatia; silvestar.meznaric@gmail.com (S.M.); darinka.vuckovic@medri.uniri.hr (D.V.); 2 Analytical Toxicology and Mineral Metabolism Unit, Institute for Medical Research and Occupational Health, 10000 Zagreb, Croatia; ibrcic@imi.hr; 3 Faculty of Health Studies, University of Rijeka, Viktora Cara Emina 5, 51000 Rijeka, Croatia; tomislav.pavlesic@fzsri.uniri.hr; 4 Department of Environmental Health, Teaching Institute of Public Health of Primorsko-Goranska County, 51000 Rijeka, Croatia; goranka.crnkovic3@gmail.com; 5 Bioinstitut d.o.o., R. Steinera 7, 40000 Čakovec, Croatia; lesar@bioinstitut.hr

**Keywords:** symbiotic, *Lactiplantibacillus*, honeydew honey, *Salmonella*

## Abstract

Honey is a natural food consisting mainly of sugars, enzymes, amino acids, organic acids, vitamins, minerals and aromatic substances. In addition to specific organoleptic properties, honey also has other components that contribute to its nutritional and health value. Proteins, vitamins, minerals, organic acids and phenolic compounds, the most variable components of honey, are predominantly responsible for its strong bioactive effect. Honeydew honey is a less known type of honey with outstanding antimicrobial and antioxidant properties that also demonstrates prebiotic effects and can promote the growth of probiotic bacteria. Foodborne illnesses can be prevented by using probiotic strains in combination with prebiotics. The aim of this study was for the first time to determine potential synergistic antimicrobial effect of fir (*Abies alba* Mill.) honeydew honey (HS) and probiotic bacteria *Lactiplantibacillus plantarum* on *Salmonella enterica* serotype Typhimurium, a primary cause of foodborne illnesses. The effect of three different samples of fir honeydew honey on the growth of *L. plantarum* in de Man, Rogosa and Sharpe (MRS) medium and the potential synergistic effect of HSs and *L. plantarum* on the growth of *S.* Typhimurium in the Brain Heart Infusion (BHI) medium were examined. The results indicate that concentrations of 1 and 5% of all three HS samples stimulate the growth and metabolic activity of *L. plantarum*, while a concentration of 10% inhibits the growth *of L. plantarum*. The concentration of 5% of all three HS and *L. plantarum* combined inhibits the growth of *S.* Typhimurium in BHI broth. Fir honeydew honey showed potential prebiotic properties and antimicrobial activity, both of which can synergistically enhance the probiotic efficacy of *L. plantarum* against *S*. Typhimurium To conclude, the combination of fir honeydew honey and *L. plantarum* represents a successful combination against *S.* Typhimurium and additional experiments are necessary regarding the mechanisms of their combined effect.

## 1. Introduction

Honey is an animal and plant-based product traditionally used for its healing and antimicrobial properties. Its health properties are dependent on the geographic origin and type of honey (botanical origin). One of the highest quality honeys, although it is still a relatively unknown type of honey, is honeydew honey—honey that honeybees (*Apis mellifera*) produce from honeydew secreted by aphids and some scale insects as they feed on plant sap. A higher content of proteins, minerals and phenolic compounds in honeydew honeys compared to nectar honeys significantly contributes to their remarkable biological activities including strong antimicrobial action [1]. 

Studies on the antibacterial activity of honeydew honeys reported superior antibacterial efficacy compared to nectar honeys, even stronger than manuka honey [2,3,4,5]. The antibacterial activity of honeydew honeys could be largely attributed to hydrogen peroxide formation [2,3,4]. However, results of recent research indicated that although hydrogen peroxide plays an important role in the inhibition of bacterial growth, phenolics and their interaction with hydrogen peroxide might be the key factors for the antibacterial activity of honeydew honeys [6].

Several studies demonstrated the prebiotic effect of honey on probiotic bacteria, which as part of the body’s natural microbiota plays an important role in maintaining health and preventing diseases by various pathogenic microorganisms in the gut [7,8]. In recent times, there has been a renewal of interest in the use of probiotics as biotherapeutic agents as scientific evidence continue to accumulate on the properties, functionality and beneficial effects of probiotic bacteria on humans. The investigation of a new probiotics is driven by the growing demand for probiotic functional food and beverages as well as dietary supplements due to growing consumer awareness regarding the concept of preventive health care. [9,10].

Various probiotic strains have been identified over the years and the most known probiotic bacteria is *Lactiplantibacillus plantarum* (former *Lactobacillus plantarum*). This probiotic strain stimulates the digestive system and fights off disease-causing bacteria by maintaining the intestinal barrier and probiotic-mediated immunomodulation [11]. Probiotic supplements are used to treat or prevent specific health problems, such as seasonal allergies and irritable bowel syndrome (IBS) [12]. The action mechanism of probiotics is not yet fully clarified; however, it is expressed by three main mechanisms: Inhibition of the growth of undesirable microorganisms in the intestinal tract by producing antibacterial substances and bacteriocins and competition for nutrients and bonding site; modification of bacterial metabolism and stimulation of the immune system. 

Additionally, previous in vitro studies suggested that honey, due to its chemical composition, could play a prebiotic role and favor the growth of lactobacilli and bifidobacteria [7,13,14,15]. 

On the other side, there are various strains of pathogenic bacteria that colonize the human digestive system. A well-known strain is *Salmonella enterica* serotype Typhimurium (*S.* Typhimurium). It is a non-typhoid serotype of *S. enterica* which is the primary cause of foodborne illnesses. *S*. Typhimurium causes acute inflammatory diarrhea that can progress to invasive systemic disease in susceptible patients leading to hospitalizations and deaths [16,17]. These diseases can be prevented by using probiotic strains in combination with prebiotics. 

Lack of data on the prebiotic potential of honeydew honey motivated us to test the antimicrobial and prebiotic activity of fir (*Abies alba* Mill.) honeydew honey and to determine whether there is a synergistic antimicrobial effect of this honey and probiotic bacteria *L. plantarum* on *S*. Typhimurium. 

## 2. Results

### 2.1. Honey Sample Analyses

Results of honey sample analyses are shown in Table 1. Microscopic analysis for morphometry of pollen grains confirmed that all the tested honey samples belonged to fir honeydew honey [18]. Their water content was within the limits according to the Codex Alimentarius and to the EU Draft 96/0114 (CNS) (≤21%) [19]. The ash content of HSs was in the range from 0.59 to 0.62%, the electrical conductivity values ranged from 1.17 to 1.22 mS/cm (Table 1), which is in accordance with legislation [19]. All three HSs had the same content of glucose (23.15 g/100 g to 24.08 g/100 g) and fructose (31.255 g/100 g to 32.27 g/100 g). We did not detect sucrose in any of the tested samples. There was no significant difference in total phenol content (225–231 mgGAE/100 g)

### 2.2. Antibacterial Effect of Fir Honeydew Honey Samples

Minimum inhibitory concentrations (MIC) and minimum bactericidal concentrations (MBC) against *S.* Typhimurium were 125–150 mg/mL and HS 1 showed a slightly weaker effect compared to the other two samples. The MIC concentrations against *L. plantarum* were 400 mg/mL and no bactericidal effect was detected (Table 2). 

### 2.3. Effect of Honeydew Samples on the Growth of Lactiplantibacillus plantarum in de Man, Rogosa and Sharpe Broth

**Figure 1:**
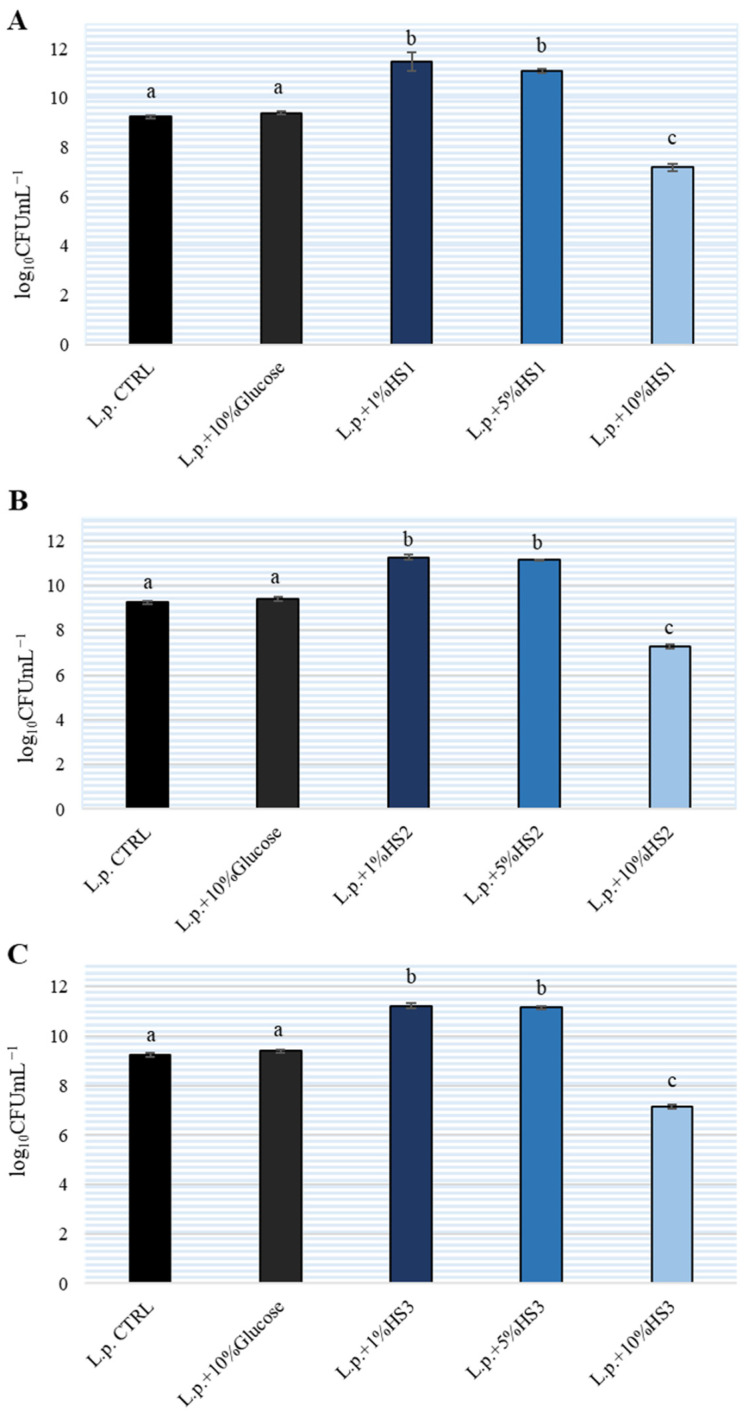
Growth of *Lactiplantibacillus plantarum* (L.p.) in de Man, Rogosa and Sharpe (MRS) broth with different honeydew honey samples (HS) (**A**–**C**) at different concentrations. The experiment was repeated three times in duplicate and the mean ± SD is shown. Different letters denote significant differences (*p* < 0.05) determined by nonparametric Wilcoxon rank-sum test.

In the experiment, the growth of *L. plantarum* in de Man, Rogosa and Sharpe (MRS) broth with three HS at concentrations of 1%, 5% and 10% was monitored after 24 h incubation. As the control, growth of bacteria in MRS broth without honeydew honey and in MRS broth with 10% glucose was determined. The results are shown on Figure 1. A comparison with the control sample (without HS) showed that *L. plantarum* grew better with the addition of 1% and 5% HS. All three HS stimulated the growth of *L. plantarum* equally well and there was no significant difference between them. In contrast, the addition of 10% HS to MRS broth resulted in an inhibition of *L. plantarum* growth by 2 logarithmic units relative to the control. Therefore, we can conclude that lower concentrations of HS will stimulate the growth of *L. plantarum* while higher concentrations (above 10%) inhibit the growth of lactobacilli. At the same time, the addition of 10% glucose to MRS broth did not affect the growth of lactobacilli in MRS broth. The addition of 5% HS to MRS broth did not result in bacterial growth after 24 h incubation and additional cultivation on Mueller Hinton (MH) agar and MRS agar indicating that no other bacteria were present in the HSs as contamination.

**Figure 2:**
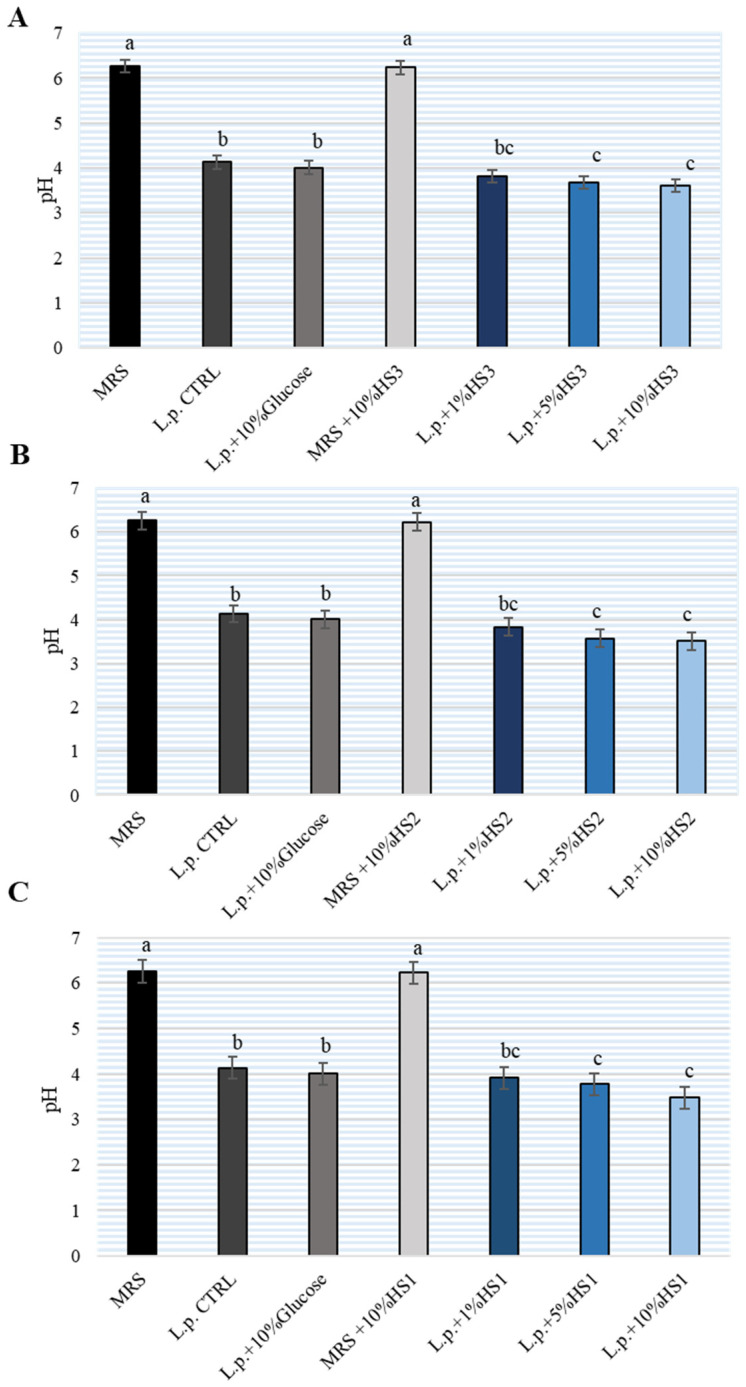
pH value of de Man, Rogosa and Sharpe (MRS) broth after cultivation of *Lactiplantibacillus plantarum* (L.p.) with the addition of different concentrations of different honeydew honey samples (HS) (**A**–**C**). The experiment was repeated two times and the mean value ± SD were shown. Different letters denote significant differences (*p* < 0.05) determined by nonparametric Wilcoxon rank-sum test.

To monitor the metabolic activity of *L. plantarum* after 24 h of incubation, the pH of MRS broth was determined (Figure 2). Bacterial-free MRS broth and MRS broth with 10% HSs had a pH of 6.2 ± 3. After 24 h of incubation of *L. plantarum* without HS, the pH was lowered (around pH4, 5 ± 2). Incubation with different concentrations of HS led to a significant decrease (pH3, 6 ± 2) in pH relative to *L. plantarum* growth without HS. No differences were observed between the three HSs samples examined. The addition of glucose to the MRS broth did not affect the further lowering of the pH. Therefore, unlike glucose supplementation, HS supplementation enhanced the metabolic activity of lactobacilli in MRS broth.

### 2.4. Combined Effect of Honeydew Samples and L. plantarum on S. Typhimurium Growth in MH Broth

**Figure 3:**
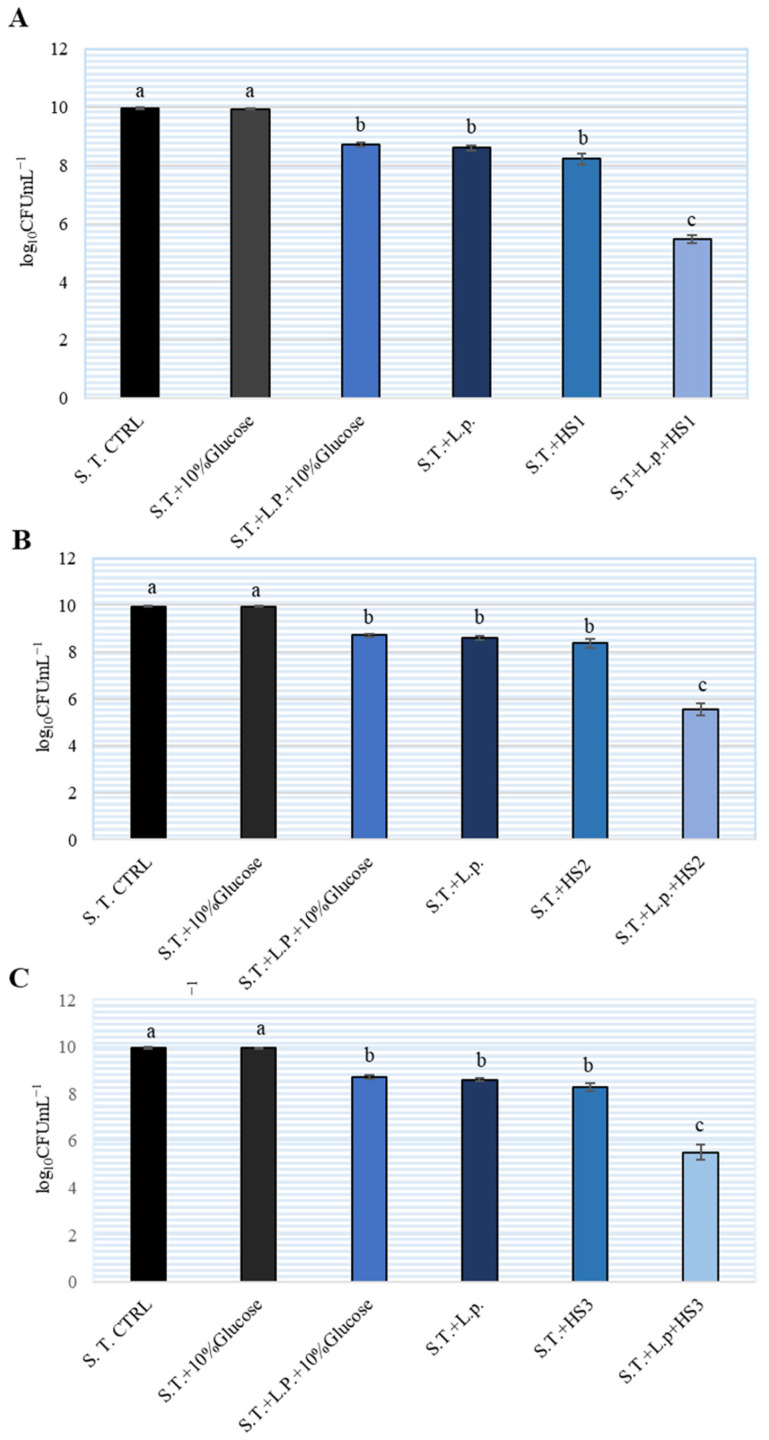
Combined inhibitory effect of different honeydew samples (HS) (**A**–**C**) and *Lactiplantibacillus plantarum* (L.p.) on the number of *Salmonella enterica* serotype Typhimurium (S.T.) in Brain Heart Infusion (BHI) broth. The experiment was repeated two times and the mean values ± SD were shown. Different letters denote significant differences between groups (*p* < 0.05) determined by nonparametric Wilcoxon rank-sum test.

To investigate the potential combined effect of HSs and lactobacilli on *S.* Typhimurium growth, MH broth and subinhibitory concentration of 5% of HS were used. The results are shown on Figure 3. The ratio of *L. plantarum* and *S.* Typhimurium was 100:1. After 24 h, we determined more than 10^10^ CFU/mL *S.* Typhimurium in MH broth (control). The addition of *L. plantarum* inhibited the increase in *Salmonella* by approximately 2 logarithmic units, while the addition of 5% HSs inhibited the increase by slightly more than two logarithms. The combination of *L. plantarum* and 5% HS inhibited the growth of *S.* Typhimurium by 5 logarithmic units. There was no statistically significant difference between the tested HSs regarding inhibition of *Salmonella* growth. The addition of glucose to the HM broth did not affect the growth of *S.* Typhimurium. The combination of 10% glucose and *L. plantarum* did not show a combined effect on the growth of *S.* Typhimurium.

**Figure 4:**
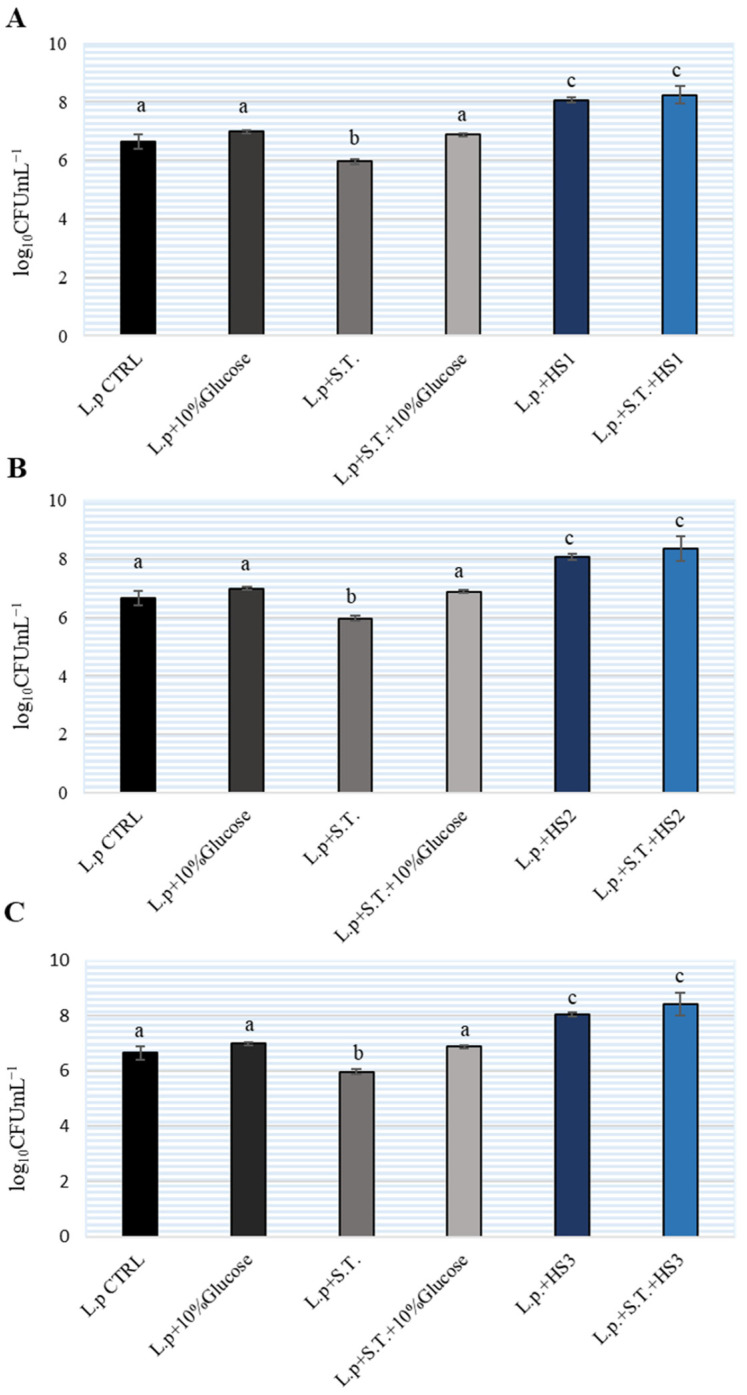
The number of *Lactiplantibacillus plantarum* (L.p.) during co-cultivation in Brain Heart Infusion (BHI) broth with different honeydew samples (HS) (**A**–**C**) and *Salmonella enterica* serotype Typhimurium (S.T.). The experiment was repeated two times and the mean values ± SD were shown. Different letters denote significant differences between groups (*p* < 0.05) determined by nonparametric Wilcoxon rank-sum test.

At the same time, the number of lactobacilli was determined in the tested samples. The results are shown on Figure 4. In MH broth and MH broth with the addition of 10% sucrose, we determined an increase of 0.5 logarithm so *L. plantarum* would not multiply successfully in MH broth. The addition of HSs significantly affected the ability of *L. plantarum* to multiply and the number of bacteria increased by 2 logarithmic units. In the sample which, in addition to lactobacilli, contained *S.* Typhimurium and HSs, the same increase by 2 logarithmic units was determined. Therefore, the addition of honey to MH broth positively affected the growth of *L. plantarum*.

## 3. Discussion

Honeydew honey is a type of honey produced from honeysuckle; a sweet substance secreted by aphids after processing plant juice. In addition to its attractive sensorial characteristics (dark brownish color and sweet flavor with pleasant, slightly resinous aftertaste and aroma), honeydew honey is valued due to its pronounced antibacterial potential. In our previous study with samples of fir (*Abies alba* Mill.) honeydew honey collected from Gorski kotar (Croatia), we proved an antibacterial effect against resistant strains of *Acinetobacter baumannii* and methicillin-resistant *Staphylococcus aureus* (MRSA) and the bactericidal effect was concentration dependent [20]. Majtan et al. have proven the bactericidal effect of Slovak honeydew honey from the floral source of *Abies* spp. against multidrug-resistant *Stenotrophomonas maltophilia* [21]. Pérez Martin et al. showed that Spanish honeydew honey from different floral origins had the capacity to inhibit Gram-positive bacteria *Micrococcus luteus* and *Staphylococcus aureus* [22]. The antimicrobial activity of Turkish honeydew honey was tested on 12 bacteria and two yeasts. The honey samples showed the highest antimicrobial activity against *Escherichia coli* O157:H7, *S. aureus* and *Listeria monocytogenes* [23]. 

In addition to the antimicrobial effect, honeydew honey has a pronounced and antioxidant effect mostly due to biologically active compounds like phenolics. Most phenolic compounds proven in honey such as, chrysin, quercetin, pinocembrin, caffeic acid and apigenin possess potential biological activity [24,25,26]. Total phenol content in our samples is consistent with the results of previous research [26,27]. Generally, larger amounts of phenolic compounds in honeydew honeys than in flower honeys have been reported in the literature [1]. Besides phenolics, which are mainly flavonoids in honeydew honeys, catalase, peroxidase, carotenoids and non-peroxide components are also responsible for its antioxidant characteristics [28].

Furthermore, due to the oligosaccharide content, honey is also recognized as a potential prebiotic. It has been proven that honey oligosaccharides can promote the growth of lactobacilli and bifidobacteria [29,30]. A comparative study involving honey oligosaccharides has demonstrated a definite prebiotic potential, which however was not as prominent as fructooligosaccharide (FOS) [31]. One of the possible superiorities of honeydew honey as a prebiotic over nectar honey may be due to the significantly higher average content of oligosaccharide melezitose. However, honeydew honey is still one of the rarer and relatively unknown types of honey, and research on its functional potential should be further explored [32,33,34].

Potentially probiotic microorganisms are extensively studied and are used in a wide range of applications such as prevention of food poisoning, treatment of certain gastrointestinal disorders, food preservation, etc. [35,36]. Lactic acid bacteria, predominantly lactobacilli, are the most frequently mentioned potentially probiotic bacteria. These bacteria have been shown to interfere with pathogenic bacteria by different mechanisms, like lowering the pH and production of antimicrobial compounds such as lactic acid, hydrogen peroxide and bacteriocin-like substances [37]. Lactic acid bacteria can inhibit the adhesion of pathogenic bacteria on intestinal epithelial cells, and consequently reduce pathogen colonization and prevent infection [38,39].

In our previous research, we isolated the *L. plantarum* strain B from homemade sheep’s cheese [39]. This strain showed metabolic activity and lowered the pH of the BHI medium with the addition of bile salts. Furthermore, during cocultivation with *S*. Typhimurium, *L. plantarum* strain B significantly inhibited *Salmonella* growth and it was shown that the mechanism of action was probably related to the lowering of pH of the media. Other authors also point out that one of the main mechanisms of action of probiotic microorganisms is lowering the pH, which is especially important in the case of *S.* Typhimurium, which is extremely sensitive to low pH [40,41]. Kajiwara et al. showed effects of honey on lactic and acetic acid production by intestinal *Bifidobacterium* spp. in a manner like those of FOS, galactooligosaccharide and inulin [42]. All of the honey types (wild and commercial, 5%) supported the growth and acid production in skim milk [43]. 

Additionally, the tested *L. plantarum* strain showed good adhesion properties on human enterocyte cell line Caco-2 and inhibited the adhesion of *S.* Typhimurium during cocultivation or after pretreatment. Another important property of probiotic bacteria is the ability to aggregate, and the *L. plantarum* strain B showed high auto-aggregation properties (≥80%) and coaggregated with *S.* Typhimurium (≥30%) [44]. Therefore, in addition to lowering the pH and aggregation property, this bacterium appears to use other mechanisms that need to be further studied. Since both honeydew honey and the tested *L. plantarum* strain showed good antibacterial properties, we wanted to examine their potential combined or synergistic effect. Table 2 indicates that it has showed an antibacterial effect on both tested bacteria, with the MIC being several times lower for *S.* Typhimurium (about 125 mg/mL). To test whether the HS tested had a potential prebiotic effect on *L. plantarum* strain B, different concentrations were tested (Figure 1) and the application of 1% and 5% HS stimulated the growth of *L. plantarum*. The addition of 10% glucose did not have any effect on the growth of *L. plantarum*. To claim that HSs possess a prebiotic effect the effect of the combination of glucose and fructose should be examined. In addition to glucose, the effect of sucrose was also examined and had no effect on lactobacilli growth (data not shown). From this we can speculate that simple carbohydrates are not the only ones responsible for stimulating the growth of *L. plantarum*. The favorable effect of honey on the growth of lactobacilli has been previously reported by several authors (Mohan et al., Shamala et al., Jiang et al.) [7,15,45]. It seems that phenolics and oligosaccharides have a synergistic effect on human intestinal microbes, which was also speculated by Jiang et al. who determined the positive impact of these compounds from buckwheat honey on the growth of *Bifidobacteria* [45]. Further research is needed to examine the effect of oligosaccharides present in this fir honeydew honey on the growth stimulation of *L. plantarum*.

To investigate the potentially combined inhibitory effect of HS and *L. plantarum* on *S.* Typhimurium, bacteria were grown in BHI broth. To study the combined effect, we chose a 5% solution of HS and the ratio of *S.* Typhimurium and *L. plantarum* was 1:100. In previous tests, we determined that this ratio of bacteria showed the best inhibition (data not shown). From the results we can see that a single application of HSs or *L. plantarum* equally inhibited the growth of *S.* Typhimurium, while their combination proved to be the most effective. To the best of the authors’ knowledge, this is the first report of a combined antimicrobial effect of honeydew honey and probiotic bacteria *L. plantarum* on *S*. Typhimurium. 

In the same experiment, we monitored the number of lactobacilli that multiply very slowly in this medium. The addition of HSs resulted in an increase in the number of lactobacilli by 2 logarithmic units and a decrease in the pH of the sample (around pH5, 3 ± 2). From the above, we could conclude that the reason for the decrease in *S.* Typhimurium in the samples lies in the fact that lactobacilli are metabolically active and that their number in the samples increased, which increased the chance of coaggregation. In this way the bacteria are in close contact and better exhibit their antimicrobial effect. Interestingly, the addition of 10% glucose did not show the same effect and did not lead to a significant increase in the number of lactobacilli in the samples, nor did it inhibit the increase in *Salmonella*. Therefore, we can say that other active components that manifest their effect are obviously present in honeydew samples.

## 4. Materials and Methods

### 4.1. The Honeydew Honey Samples

The fir (*Abies alba* Mill.) honeydew samples (HS) were purchased from Gorski d.o.o., Fužine, Croatia. They were obtained during summer 2015 from the mountain region Gorski kotar (western part of Croatia) defined by Universal Transverse Mercator (UTM) system coordinates as follows: Sample 1 (HS 1), location 1: Lič; Potkoš (45°17′59″ N, 14°44′12″ W), sample 2 (HS 2), location 2: Crni lug; Lazac (45°22′02″ N, 14°43′10″ W) and sample 3 (HS 3), location 3: Fužine; Vrelo (45°19′34″ N, 14°42′16″ W). HS were stored at 4 °C in hermetically closed glass jars until analysis. For microbiological analyses, HS samples were diluted in MRS or BHI broth and pasteurized at 70 °C for 15 min [46].

### 4.2. The Honeydew Honey Analyses

The melissopalynological analysis followed the methods recommended by the International Commission for Bee Botany (now known as International Commission on Plant Pollinator Relations; ICPPR) [47]. Microscopic analysis for morphometry of pollen grains and honeydew elements such as hyphae, fungal spores, mycelium or unicellular algae was performed on a Hund H500 (Helmut Hund GmbH, Wetzlar, Germany) light microscope with attached digital camera (model Dino-Eye AM423U; Dino-Lite, AnMo Electronics Corp., Hsinchu, Taiwan) and coupled to an analysis system (DinoCapture 2.0 v. 1.4.9; Dino-Lite). Water content was determined by refractometry, measuring the refractive index, using standard model Abeé refractometer (Carl Zeiss, Jena, Germany) at 20 °C. Water content (%) was obtained from the Chataway table [48]. Electrical conductivity was measured in a solution of 20 g honey sample in low-conductivity water system at 20 °C using a conductometer (HI-8733; Hanna Instruments, Woonsocket, RI, USA), while the ash content was calculated according to the results of electrical conductivity [13]. To determine concentrations of sugars (glucose, fructose, sucrose) in HS, high performance liquid chromatography combined with RI detector (HPLC-RID) (Knauer, Berlin, Germany) was used [48]. 

The Folin–Ciocalteu method was used to determine the concentration of total phenols [49]. Gallic acid (Sigma Aldrich, Darmstadt, Germany) was used as a standard to produce the calibration curve. 

Each HS (1 g) was diluted in 10 mL distilled water and filtered through Whatman No. 1 paper. This solution (0.5 mL) was then mixed with 2.5 mL of 0.2 N Folin–Ciocalteu reagent (Sigma Aldrich, Darmstadt, Germany) for 5 min and 2 mL of 75 g/L sodium carbonate (Na_2_CO_3_) (Sigma Aldrich, Darmstadt, Germany) was then added. After incubation at room temperature for 2 h, the absorbance of the reaction mixture was measured at 760 nm against a methanol blank (Eppendorf Biofotometar, Hamburg, Germany). The mean of three readings was used and the total phenolic content was expressed in mg of gallic acid equivalents (GAE)/100 g of honey.

### 4.3. Bacterial Strains and Growth Conditions

*L. plantarum* isolates strain B from homemade sheep’s cheese were kindly provided by Prof. Jadranka Frece from the Faculty of Food Technology and Biotechnology, University of Zagreb, Croatia [44,50]. *S. enterica* serotype Typhimurium ATCC 14,028 were obtained from the culture collection of the Department of Microbiology and Parasitology, Faculty of Medicine, University of Rijeka. All of the tested bacteria were kept in 30% glycerol broth at –80 °C. *L. plantarum* was grown in MRS broth (Biolife Italiana, Milan, Italy) in microaerophilic atmosphere (5% CO_2_) for 48 h at 37 °C. *S.* Typhimurium was cultivated in nutrient broth (Biolife Italiana, Milan, Italy) and the number of bacteria was determined by plate counting on Salmonella Shigella (SS) agar (Biolife Italiana, Milan, Italy) or Mueller Hinton agar (MHA) (Biolife Italiana, Milan, Italy). The number of bacteria in the suspension was determined photometrically at λ = 600 nm and the absorbance (A) was set to 0.3, corresponding to a concentration of 10^8^ CFU/mL.

### 4.4. Antibacterial Activity Assay 

MIC and MBC of the HSs were determined using a standard microdilution technique in Muller Hinton Broth (MHB). Each honey sample was dissolved in MHB to prepare stock solutions of 0.8 g/mL. Furthermore, twofold serial dilutions in MHB were prepared from stock solutions of each honey sample to give final concentrations ranging from 0.0125 to 0.4 g/mL. A volume of 100 µL of each diluted sample was mixed with equal volume of bacterial suspension. Positive (broth and inoculum) and negative (simple broth) growth controls were prepared. The plates were incubated for 24 h at 37 °C and 120 rpm (Unimax 1010; Heidolph Instruments GmbH&CO., KG, Schwabach, Germany). MIC values were taken as the lowest concentration of honey sample that produced no visible bacterial growth compared to the control wells after 24 h of incubation at 37 °C. MBC is determined by inoculating the samples used for MIC determinations onto MHA and incubating further for 18–24 h. MBC was defined as the lowest concentration of honey sample that killed ≥99% of bacteria. Meropenem for *S.* Typhimurium and gentamicin for *L. plantarum* served as positive controls of growth inhibition. The final antibiotic concentrations used in the assays ranged between 0.0015 and 3.84 mg/L for both antibiotics. The results were interpreted following EUCAST recommendations [51]. 

### 4.5. Effect of Honeydew Samples on the Growth of L. plantarum in MRS Broth

The suspension of lactobacilli (10^6^ CFU/mL) in MRS broth was inoculated using different concentrations of honeys (1, 5 and 10%). After incubation for 24 h at 37 °C and 120 rpm (Unimax 1010; Heidolph Instruments GmbH & CO., KG, Schwabach, Germany), the number of lactobacilli was determined by plating ten-fold dilutions on MRS agar. The ability of the isolates to reduce the pH of the medium was tested (Mettler Toledo pH Meter).

### 4.6. Effect of Co-Cultivation of L. plantarum and Honeydew Samples on S. Typhimurium Growth

To investigate the potentially synergistic effect of *L. plantarum* and HSs on *S.* Typhimurium growth, the concentration of HSs used was 5%. A bacterial suspension (*S.* Typhimurium) at a concentration of 10^4^ CFU/mL and a suspension of lactobacilli at a concentration of 10^6^ CFU/mL in BHI broth were prepared. A bacterial suspension of *S.* Typhimurium without the addition of HS and lactobacilli was used as the control. The individual effect of HS and the combined effect of HS and lactobacilli on *S.* Typhimurium growth were tested. In addition, the effect of glucose (10%) was tested individually and in combination with lactobacilli. The number of *S.* Typhimurium was monitored by plating tenfold dilutions of samples on *Salmonella Shigella* (SS) agar. The pH of the samples was also monitored at the end of the experiments.

### 4.7. The Number of L. plantarum during Co-Cultivation 

The number of lactobacilli was monitored during the previously described experiment (Section 4.6). The number of lactobacilli was determined by plating ten-fold dilutions on MRS agar.

### 4.8. Statistical Analysis

Statistical analysis was preformed using analytic software Statistica 13.5.0.17. (TIBCO, Palo Alto, CA, USA). Statistical significance was tested on a significance level of *p* < 0.05 by nonparametric Wilcoxon rank-sum test and verified with Mann–Whitney U test. Letters on top of columns present statistical significances. Results were graphically displayed using Microsoft Excel.

## 5. Conclusions

The combined combination of fir honeydew honey and probiotic bacteria *Lactiplantibacillus plantarum* can be more beneficial in inhibiting *S.* Typhimurium growth than their individual application. Fir honeydew honey shows potential prebiotic properties, with antimicrobial activity, both of which can enhance the probiotic efficacy of *L. plantarum* against *S.* Typhimurium.

## Figures and Tables

**Table 1:** Characterization of fir (*Abies alba* Mill.) honeydew samples (HSs).

Samples	El. Conductivity/ (mS/cm)	w(Water)/ %	w(Ash)/ %	Glucose/ g/100g	Fructose/ g/100g	Sucrose/ g/100g	Total Phenols/ mgGAE/100g
HS 1	1.22	19.1	0.62	23.15	31.25	-	231
HS 2	1.17	18.4	0.59	24.08	32.27	-	228
HS 3	1.22	17.7	0.62	23.45	32.15	-	225

**Table 2:** Minimum inhibitory concentration (MIC) and minimum bactericidal concentration (MBC) of fir honeydew honey (*Abies alba* Mill.; HS 1–3) against *Salmonella enterica* serotype Typhimurium and *Lactiplantibacillus plantarum*.

Bacteria	*S.* Typhimurium		*L. plantarum*	
Samples	MIC (mg/mL)	MBC (mg/mL)	MIC (mg/mL)	MBC (mg/mL)
HS 1	150 ± 50	175 ± 43.3	400 ± 0.0	>400
HS 2	125 ± 43.3	125 ± 43.3	400 ± 0.0	>400
HS 3	125 ± 43.3	125 ± 43.3	400 ± 0.0	>400
Meropenem	0.06 ± 0.0	0.06 ± 0.0	ND	ND
Gentamicin	ND	ND	0.004 ± 0.0	0.004 ± 0.0

## Data Availability

The data that support the findings of this study are available from the corresponding author upon reasonable request.
